# Oxygen drives benthic-pelagic decomposition pathways in shallow wetlands

**DOI:** 10.1038/s41598-017-15432-3

**Published:** 2017-11-08

**Authors:** Gea H. van der Lee, Michiel H. S. Kraak, Ralf C. M. Verdonschot, J. Arie Vonk, Piet F. M. Verdonschot

**Affiliations:** 1 Institute for Biodiversity and Ecosystem Dynamics (IBED-FAME), University of Amsterdam, P.O. Box 94240, 1090 GE Amsterdam, The Netherlands; 2 Wageningen Environmental Research, Wageningen UR, P.O. Box 47, 6700 AA Wageningen, The Netherlands

## Abstract

Oxygen availability is perceived as an important environmental factor limiting POM decomposition. In shallow wetlands, however, the impact of commonly observed anoxic conditions in the benthic layer on the relative contribution of microbes and invertebrates to POM decomposition remains largely unknown. Therefore, the aim of this study was to determine if dissolved oxygen drives benthic-pelagic decomposition pathways in shallow wetlands. Dissolved oxygen concentration, invertebrate community composition, microbial decomposition and invertebrate consumption were measured in the benthic and pelagic layer of 15 permanent drainage ditches. We showed that an increased duration of anoxic conditions in the benthic layer of the ditches was related to increased microbial decomposition in this layer, while invertebrate consumption decreased in the benthic layer and increased in the pelagic layer. The increased invertebrate consumption in the pelagic layer was related to the presence of amphipods. We concluded that anoxic conditions in the benthic layer of shallow wetlands relate to an increase in microbial decomposition and a decrease in invertebrate consumption, as detritivorous invertebrates move to the pelagic layer to consume particulate organic matter. This illustrates that environmental conditions, such as dissolved oxygen, may drive the relative importance of aquatic organisms to ecosystem functioning.

## Introduction

Dead particulate organic matter (POM) fuels shallow wetland food webs by serving as a food source for interacting microbes and invertebrates^1,2^. The rate of POM decomposition is therefore influenced by microbial and invertebrate community composition, but also by POM quality, and by the physicochemical environment^3–5^. Especially, oxygen availability has been proposed an important environmental factor limiting POM decomposition^3,6^. In shallow wetlands, considerable differences in daily and seasonal dissolved oxygen concentrations have been measured along depth gradients in the water column^7–10^. This may alter the relative importance of microbes and invertebrates for POM decomposition in each layer, as they are differently adapted to function under low dissolved oxygen conditions^3,11^.

Dissolved oxygen concentrations are the result of whole-system primary production (release of O_2_) and respiration (uptake of O_2_)^12^. In shallow wetlands, anoxic conditions typically occur in the benthic layer from late spring through summer, as the oxygen demand is high at the bottom due to POM processing, in combination with stratification of the water column due to high water temperatures and decreased water movements^7,13^. Despite the differences in oxygen conditions between the benthic and pelagic layer, POM decomposition in shallow wetlands is pre-dominantly studied in the benthic layer^13–15^. However, a large amount of plant biomass already decomposes in a standing-dead position in the pelagic layer before the shoot material collapses to the benthic layer^16^. Furthermore, algal litter in the pelagic layer can also form an important food source in eutrophic shallow wetland food webs^17^. Hence, in shallow wetlands POM decomposition takes place in both the benthic and pelagic layer, yet under varying oxygen conditions.

It is generally believed that anoxic conditions slow down invertebrate consumption rates by lowering invertebrate densities^3,6^. Some aquatic invertebrate species are killed or suffer from sub-lethal effects under anoxic conditions^18,19^, while mobile organisms may move to the pelagic layer^20^. Furthermore, it has been suggested that anoxia slows down microbial decomposition rates, as oxygen is energetically the most favourable electron acceptor^21^. Data from microbial litter breakdown studies do, however, not always support the assumption that decomposition proceeds more rapidly under aerobic than anoxic conditions^22,23^. A diverse assemblage of microbial functional groups coexists in freshwater sediments and water, and depending on the prevailing conditions one or the other functional group may become active and dominant^24,25^. Thus, the relative contribution of the interacting microbes and invertebrates to POM decomposition and consumption in the benthic and pelagic layer of shallow wetlands remains largely unknown.

The aim of this study was therefore to determine if dissolved oxygen drives benthic-pelagic POM decomposition pathways in shallow wetlands. We hypothesized that low dissolved oxygen concentrations in the benthic layer I) do not change microbial decomposition rates, as other functional groups adapted to low oxygen become active and dominant^24^, and II) lead to lower invertebrate consumption, as they die^18,19^ or move to the pelagic layer^20^. To test these hypotheses, we performed a field study in which we continuously monitored dissolved oxygen concentrations, determined invertebrate community composition, and quantified microbial decomposition and invertebrate consumption in the benthic and pelagic layer of 15 permanent peat drainage ditches in The Netherlands.

## Results

### Dissolved oxygen conditions

**Figure 1:**
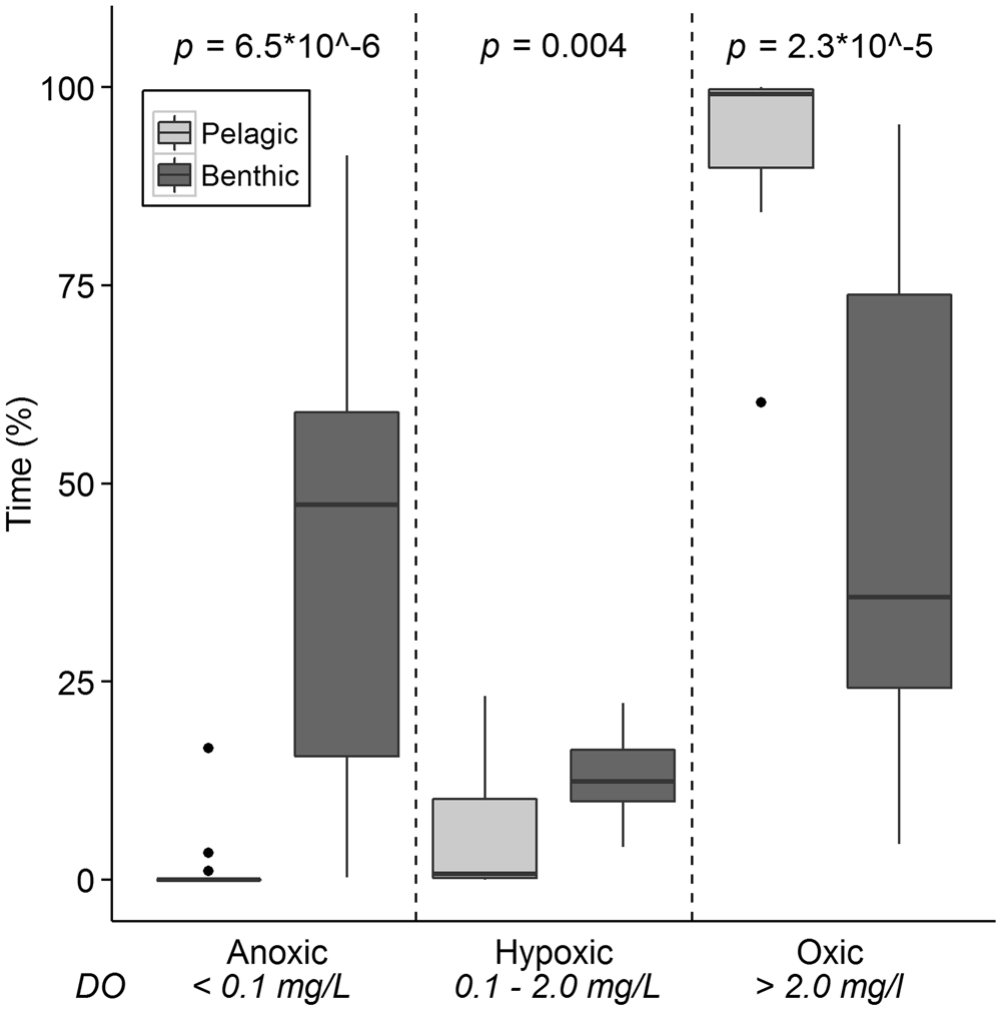
Boxplot of dissolved oxygen (DO) conditions during 55 days of measurements in the pelagic and benthic layer. Boxes are inter-quartile ranges (25^th^ percentile to 75^th^ percentile); whiskers extend to 1.5 * IQR; dots are outliers. p-values indicate statistical difference between the benthic and pelagic layer are for percent time anoxic, hypoxic and oxic (Mann-Whitney pairwise comparisons; n = 15).

The duration of anoxic, hypoxic, and oxic conditions in the 15 ditches during the 55 days of measurements differed significantly (all *p* < 0.01) between the benthic and pelagic layer (Fig. 1). The pelagic layer was almost always oxic in all ditches (mean percent of time ± sd = 93 ± 11%), and anoxia and hypoxia were rarely observed. The benthic layer showed large variations in dissolved oxygen conditions with ranges between the ditches from 0 to 91% of the time anoxic, 4 to 22% of the time hypoxic, and 4 to 95% of the time oxic. The percent of time that the hypoxic and anoxic conditions occurred in the benthic layer was significantly higher than in the pelagic layer (*p* = 0.004 and *p* = 2.3 * 10^−5^, respectively). Dissolved oxygen concentrations did not relate significantly to water temperature in the benthic layer (R^2^ = 0.08, *p* = 0.30), or the pelagic layer (R^2^ = 0.05, *p* = 0.44).

### Invertebrate community composition

A total of 2857 individuals belonging to 79 taxa were caught in the activity traps. The detritivores comprised 63.9% of the total invertebrate abundance. The four most dominant (>10% of the detritivore abundance) detritivore taxonomic groups were corixids, amphipods, oligochaetes, and isopods, which constituted 30.0%, 29.4%, 13.9%, and 11.2% to the total detritivore abundance, respectively.

Significant differences were observed between the overall invertebrate richness and abundance in the benthic and pelagic layer (all *p* < 0.05; Table 1). There was, however, no significant difference in detritivore richness between the layers (*p* = 0.06). Detritivores were significantly more abundant in the benthic layer (mean individuals ± sd = 72 ± 42) than in the pelagic layer (mean individuals ± sd = 50 ± 31, *p* = 7.72 * 10^−3^), which was reflected by significantly higher number of corixids and isopods in the benthic layer. Abundance of amphipods and oligochaetes did not differ significantly between the benthic and pelagic layer.

**Table 1:** Richness and abundance of invertebrates caught in the benthic and pelagic layer. Statistical difference in richness (number of taxa) and abundance (number of individuals) between the benthic and pelagic layer are presented (paired sampled t-test; n = 15, mean ± sd).

Invertebrate metric		Benthic layer	Pelagic layer	*p*
Richness (number of taxa)	Overall	19.3 ± 7.1	15.7 ± 3.9	0.03
	Detritivores	9.5 ± 3.2	7.8 ± 1.9	0.06
Abundance (number of individuals)	Overall	109.3 ± 62.6	81.2 ± 31.0	0.02
	Detritivores	72.1 ± 42.1	49.5 ± 31.2	7.72 * 10^−3^
	Corixids	31.1 ± 28.8	8.1 ± 7.2	1.74 * 10^−5^
	Amphipods	14.7 ± 12.4	21.1 ± 24.1	0.12
	Oligochaetes	5.1 ± 7.7	9.0 ± 10.7	0.06
	Isopods	12.4 ± 15.2	3.7 ± 5.3	1.35 * 10^−3^

### Microbial decomposition and invertebrate consumption

**Figure 2:**
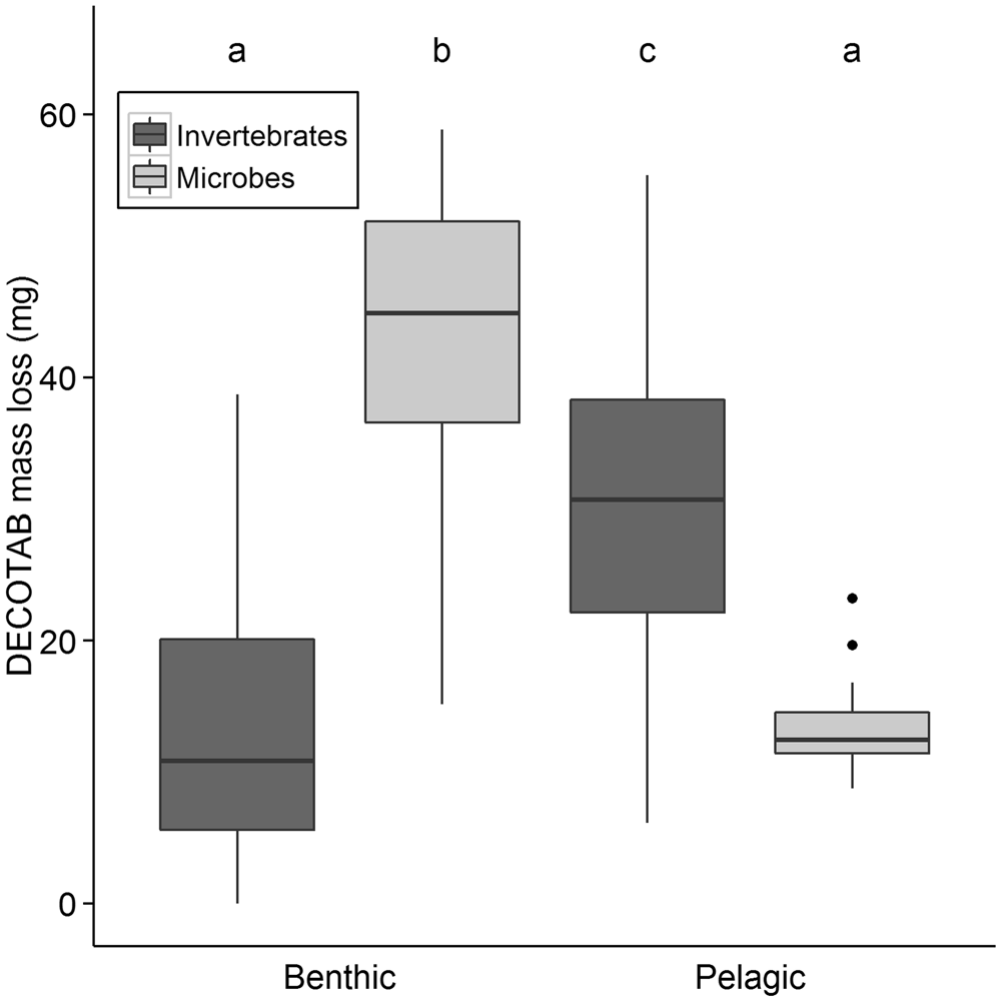
Boxplot of loss of DECOTAB mass (mg) after 55 days of exposure in the benthic and pelagic layer, expressed as invertebrate consumption and microbial decomposition. Boxes are inter-quartile ranges (25^th^ percentile to 75^th^ percentile); whiskers extend to 1.5 * IQR; dots are outliers. Different letters indicate statistical difference between DECOTAB mass loss (Kruskal–Wallis test and post-hoc Mann-Whitney pairwise comparisons; n = 15).

The contribution of microbial decomposition and invertebrate consumption to DECOTAB mass loss was significantly different between the benthic and pelagic layer during the 55 days of the measurements (*p* = 3.5 * 10^−7^; Fig. 2). In the benthic layer the microbial decomposition (mean mass loss ± sd = 42 ± 13 mg) was higher than invertebrate consumption (mean mass loss ± sd = 14 ± 11 mg), while the opposite was observed in the pelagic layer where invertebrate consumption (mean mass loss ± sd = 31 ± 13 mg) was higher than microbial decomposition (mean mass loss ± sd = 14 ± 4 mg).

### Effect of anoxic conditions in the benthic layer

**Figure 3:**
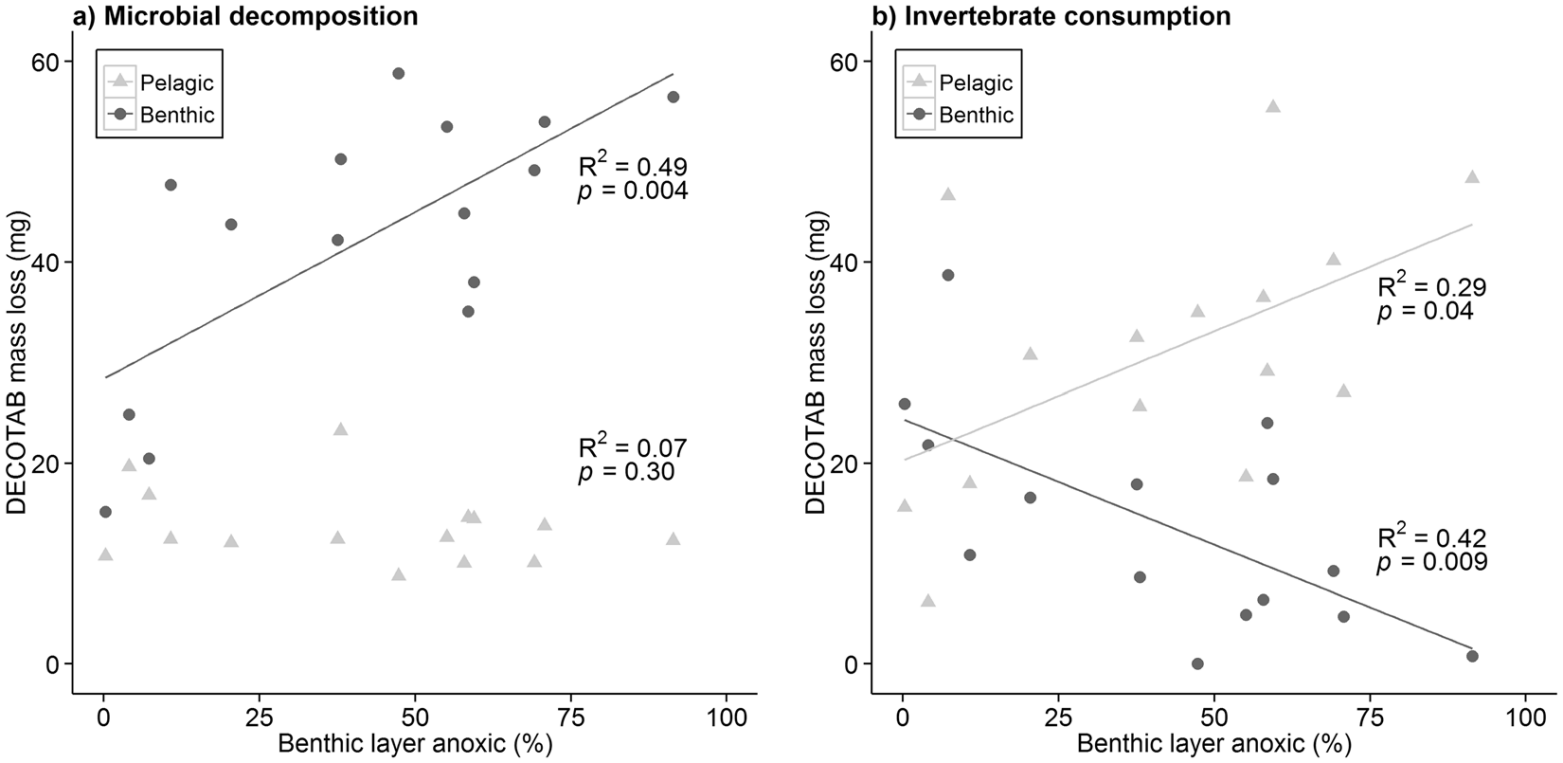
Relation between anoxic conditions (DO < 0.2 mg/L) in the benthic layer (% of time) and loss of DECOTAB mass (mg) after 55 days of exposure in the benthic and pelagic layer for (**a**) microbial decomposition, and (**b**) invertebrate consumption (linear regression; n = 15).

An increase in anoxic periods in the benthic layer related to a significant increase in microbial decomposition in the benthic layer (R^2^ = 0.49, *p* = 0.004), but showed no relation to the microbial decomposition in the pelagic layer (Fig. 3a). Invertebrate consumption significantly decreased in the benthic layer (R^2^ = 0.42, *p* = 0.009), and significantly increased in the pelagic layer (R^2^ = 0.29, *p* = 0.04) in relation to increasing time that the benthic layer was anoxic (Fig. 3b). Ditches that were not or for limited time anoxic in the benthic layer showed similar microbial decomposition and invertebrate consumption in the benthic and pelagic layer (compare Fig. 3a,b).

### Detritivorous invertebrates and consumption

**Figure 4:**
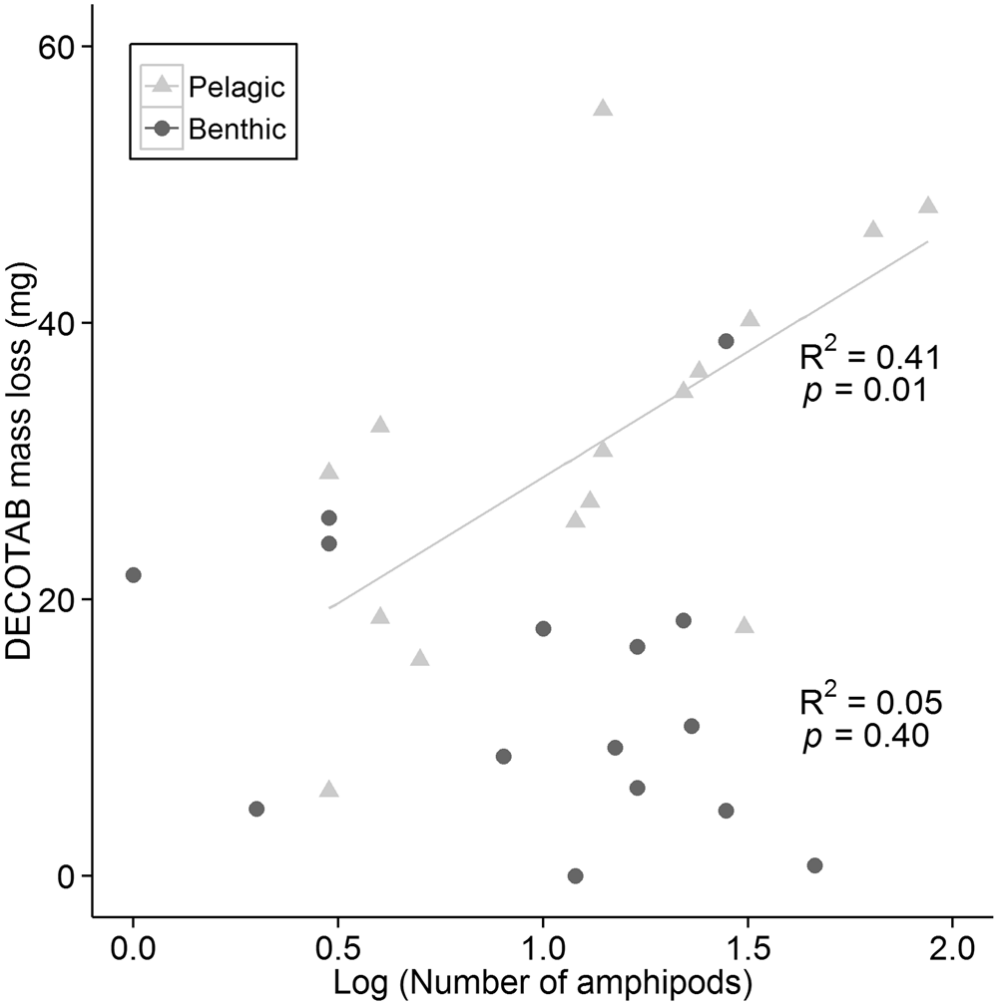
Relation between number of amphipods (log_10_(x + 1)) and loss of DECOTAB mass (mg) after 55 days of exposure in the benthic and pelagic layer (linear regression; n = 15).

The number of amphipods (97% *Gammarus pulex* and 3% *Crangonyx pseudogracilis*) caught in the traps related significantly to the invertebrate consumption of the DECOTABs in the pelagic layer (R^2^ = 0.41, *p* = 0.01), but not in the benthic layer (R^2^ = 0.05, *p* = 0.40; Fig. 4). No significant relation was observed between invertebrate consumption and the number of corixids (pelagic R^2^ = 0.01, *p* = 0.71; benthic R^2^ = 0.01, *p* = 0.68), number of oligochaetes (pelagic R^2^ = 0.00, *p* = 0.86; benthic R^2^ = 0.07, *p* = 0.33), or the number of isopods caught in the traps of each layer (pelagic R^2^ = 0.08, *p* = 0.32; benthic R^2^ = 0.00, *p* = 0.82).

## Discussion

We determined microbial decomposition and invertebrate consumption in permanent drainage ditches, characterized by different dissolved oxygen conditions in the benthic layer compared to the pelagic layer. The pelagic layer was almost always oxic in all drainage ditches, while the benthic layer covered the entire oxic-anoxic range. Similar dissolved oxygen patterns have been observed in shallow wetland environments other than drainage ditches^9,26^. Our results showed that an increased duration of anoxic conditions in the benthic layer related to increased microbial decomposition (rejecting our first hypothesis), while invertebrate consumption simultaneously decreased in this layer (accepting our second hypothesis).

Longer time periods of anoxic conditions in the benthic layer related to higher microbial decomposition rates. Faster POM breakdown at low oxygen concentrations has also been reported for lakes^22,23^. It was, however, not possible to determine whether low dissolved oxygen concentrations were the cause or the consequence of the high microbial activities in the benthic layer, as under anoxic conditions oxygen production and consumption rates cannot be directly derived from dissolved oxygen concentrations. Primary production rates of epipelon and epiphyton can be high in the benthic layer of shallow wetlands^27^, but this oxygen may be immediately used by the microbial community, still leading to anoxic conditions. Under complete oxic conditions in the benthic and pelagic layer decomposition rates were similar, so we assume that under anoxic conditions in the benthic layer additional functional groups of bacteria have become metabolically active, including denitrifiers, manganese-iron reducers, sulphate reducers, or fermenters^24^. Adaptation of these microbial functional groups to daily and seasonal anoxic conditions, in combination with excess availability of an alternative electron acceptor (e.g. nitrate), may thereby enhance microbial decomposition in shallow wetlands^13^. Such changes in the microbial community can occur abruptly between oxic and anoxic states^25^. However, other processes that influence microbial decomposition rates may also have changed under anoxic conditions in the benthic layer, such as an increased release of compounds from the sediments needed for decomposition^28^, or differences in numbers and diversity of organisms that graze upon microbes^29^ and periphytic algae, which may have stimulated microbial decomposers in breaking down POM^30^. No effect of anoxic conditions in the benthic layer on the microbial decomposition in the pelagic layer was observed, which confirms the idea that microbial functional activity corresponds to small-scale variations in chemical conditions^24^, in this case presumably the widely varying oxygen conditions over a water column height of less than one meter.

Invertebrate consumption decreased in the benthic layer and increased in the pelagic layer as the benthic layer was anoxic for a longer time period. This suggests that the invertebrates moved higher up in the water column to avoid prolonged anoxic conditions. Yet, we observed that the detritivores remained present in the benthic layer under low oxygen conditions, which implies that they were present, but did not consume POM. Kolar & Rahel showed that in the absence of predators invertebrates moved to the pelagic layer as benthic dissolved oxygen concentrations declined, but in the presence of fish most taxa remained in the benthic layer and slowed down their activity^20^. Further, larvae of four species of caddisfly^31^ and the amphipod *Gammarus pulex* ^32^ reduced or even stopped POM consumption under low oxygen conditions. These findings are coherent with the conclusion by Verdonschot & Verdonschot that most invertebrate taxa are capable to survive a certain period of anoxia in drainage ditches, but that such events can have negative impact on their functioning (e.g. emergence and recruitment)^33^. The increase in invertebrate consumption in the pelagic layer only related to increased activity of amphipods (mainly *Gammarus pulex*) in this layer. Although functional feeding groups are often treated as one guild, sharing specific traits^34^, in reality detritivorous invertebrates have different abilities to consume POM and this capacity may alter under anoxic stress^31^. Similar to our study, Tiegs *et al*. showed that the extremely mobile and very effective leave shredder *Gammarus fossarum* was a key player in the decomposition processes in their leaf litter bag experiment in streams^35^. Gammarids were 100 to 200 times more abundant on leaf litter packs than in areas adjacent to these packs^36^. We thus suggest that mobile invertebrates, such as gammarids, take refuge in the benthic layer from predators while reducing their detritivorous activity due to low oxygen concentrations, and that they migrate to the pelagic layer to consume POM under oxic conditions.

To conclude, anoxic conditions in the benthic layer of shallow wetlands relate to an increase in microbial decomposition, and a decrease in invertebrate consumption in this layer as detritivorous invertebrates move to the pelagic layer to consume particulate organic matter. Oxygen may thus drive benthic-pelagic decomposition pathways in shallow wetlands, which illustrates that environmental conditions determine the relative importance of groups of aquatic organisms to ecosystem functioning.

## Methods

### Study area

The present field study was performed in 15 permanent peat drainage ditches located in an extensive agricultural area near Tienhoven, The Netherlands (52°09′N - 52°10′N; 5°05′E - 5°06′E). Drainage ditches are linear water bodies with negligible water movement (0–5 cm/s). The ditches were selected based on similar width (3.7 ± 1.0 m) and depth (0.6 ± 0.1 m). Physicochemical characteristics of the water column and sediment from the ditches were determined three times between May and July 2016 (methods in Supplementary material 1; results in Table 2). Water temperature, dissolved oxygen and decomposition were measured over 55 days between 26 May and 20 June 2016. Each measurement was performed in the benthic layer (<10 cm above the sediment) and in the pelagic layer (<10 cm below water surface).

**Table 2:** Overview of the physicochemical characteristics of the water column and sediment in the ditches (n = 15, mean ± sd).

Water column								Sediment		
Water temp (°C)	Conductivity (μS/cm)	Tot C (mg/L)	DOC (mg/L)	Tot N (mg/L)	${{\rm{PO}}}_{4}^{3-}$ (mg/L)	${{\rm{SO}}}_{4}^{2-}$ (mg/L)	Cl (mg/L)	C:N ratio	Tot P (mg/g)	Organic matter (%)
8 ± 0.6	291 ± 72	44.6 ± 9.7	20.9 ± 9.3	1.3 ± 0.6	0.06 ± 0.02	6.7 ± 4.7	16.9 ± 10.4	14.5 ± 1.1	0.6 ± 0.3	17.2 ± 12.8

### Dissolved oxygen conditions

In each ditch, dissolved oxygen concentration and water temperature were measured every ten minutes during 55 days with HOBO® Dissolved oxygen loggers U26-001 (Onset Computer Corporation). Loggers were placed 10 cm (pelagic layer) under the water surface and 4 cm above the bottom sediments (benthic layer). A reading of 0.2 mg/L or lower was considered to be anoxic, a reading between 0.2 and 2.0 mg/L hypoxic, and a reading of 2.0 mg/L and higher as oxic^37^. For each ditch the percent time anoxic, hypoxic and anoxic was calculated for the benthic and pelagic layer.

### Invertebrate community composition

Invertebrate community composition was determined using activity traps^38^. The traps were deployed 10 cm under the water surface (n = 5 per ditch) and on the bottom substrate (n = 5 per ditch) for one week, both at the start and at the end of the field experiment. The traps were carefully retrieved from the water, the contents poured through a sieve (250 μm mesh), and thereafter washed into a bottle with 70% ethanol. In the laboratory, the collected invertebrates were identified to the lowest practical taxonomic level, except for Tricladida, Hirudinea, and Oligochaeta which were not identified further. Bias caused by differences in taxonomic resolution (family, genus, and species) was reduced by applying a conservative adjustment procedure^39^. We defined taxa as detritivore based on a combination of the primary functional feeding group (i.e. collector gatherer, shredder or filter feeder) and food type (i.e. detritus or dead plants) based on the trait-database by Tachet *et al*.^40^ supplemented with additional literature^41,42^ (complete list of detritivores is provided in Supplementary material 2). The invertebrate data of the traps deployed at the start and the end of the experiment were aggregated per ditch (total n = 10 per ditch) before further analyses were conducted.

### Microbial decomposition and invertebrate consumption

Decomposition rates were measured using standard substrates, the Decomposition and Consumption Tablets (DECOTABs) (www.ibed.uva.nl/decotab)^43^. The DECOTABs were prepared by boiling 20 g/L of purified agar dissolved in deionized water for 3 minutes. The mixture was cooled down under continuous stirring to 60 °C at which point 60 g/L of powdered cellulose and 60 µmol/L ascorbic acid as were added. The mixture was then poured into polycarbonate moulds (15 mm diameter; 884 mm^3^ volume), and after cooling the DECOTABs were removed from the moulds and stored at 7 °C. Initial DECOTAB dry mass (70 °C, 2 days) was determined from a subset of 70 DECOTABs.

To facilitate retrieval from the field, we deployed cages (height 2 cm; diameter 10 cm) containing three DECOTABs closed off by either fine mesh (width 51 µm) to quantify decomposition by microcrobes, or coarse mesh (width 4 mm) to quantify the joint microbial decomposition and invertebrate consumption. Mass loss by leaching of compounds from the DECOTABs under controlled conditions was negligible^15^. The cages were deployed 10 cm under the water surface (fine cages n = 5 per ditch; coarse cages n = 5 per ditch) and on the bottom substrate (fine cages n = 5 per ditch; coarse cages n = 5 per ditch). After 55 days of exposure, the cages were retrieved, DECOTABs were rinsed, dried in a stove (70 °C, 2 days), and weighed. Mass loss was calculated as mean initial weight minus individual weight after exposure in the field. Microbial decomposition was defined as the mass loss of DECOTAB in the fine cages. Consumption by invertebrates was calculated by subtracting mass loss of DECOTAB in fine mesh cages from mass loss of DECOTAB in the coarse mesh cages. The decomposition rates of the five replicates per ditch were averaged for further analysis, excluding outliers according to the Dixon’s Q test^44^.

### Statistical analysis

Significant differences among oxygen conditions (percent of time anoxic, hypoxic, and oxic) in the benthic and pelagic layer were analysed using Mann-Whitney pairwise comparisons, because of deviations from homogeneity and normality of variances. To exclude the effect of water temperature as confounding factor in our experiment, water temperature was related to the mean oxygen concentration in each layer using a linear regression. To meet assumptions of normality, invertebrate data were log_10_ (x + 1)-transformed. Differences in the invertebrate community composition between the benthic and pelagic layer were compared using a paired sampled t-test. Microbial decomposition (i.e. mass loss in fine mesh cages) and invertebrate consumption (i.e. mass loss in coarse cages minus microbial decomposition) in the benthic and pelagic layer were compared using Kruskal–Wallis tests, because of deviations from homogeneity of variances. Post hoc testing was performed using Mann-Whitney pairwise comparisons (Bonferroni corrected: 0.05/3, a = 0.017) to compare the 4 groups. To assess the effect of stratification of the water column on decomposition pathways, microbial decomposition and invertebrate consumption were related to the percent time the benthic layer was anoxic using linear regression analysis. A linear regression analysis was further used to relate the dominant detritivore invertebrate groups to invertebrate decomposition. Data analyses were performed in R version 3.1.0.

### Data availability statement

The data that support the findings of this study are available from the corresponding author upon reasonable request.

## Electronic supplementary material

Supplementary material
