# Supplementary material for: Oxygen drives benthic-pelagic decomposition pathways in shallow wetlands
Source: Sci Rep. 2017 Nov 8;7:15051. doi: 10.1038/s41598-017-15432-3 (PMC5678150; doi:10.1038/s41598-017-15432-3)
Supplement: Supplementary file 1 — Supplementary material [file 41598_2017_15432_MOESM1_ESM.pdf]

Title

Oxygen drives benthic and pelagic decomposition pathways in shallow wetlands

Author names and affiliations

Gea H. van der Lee, Michiel H.S. Kraak, Ralf C.M. Verdonschot, J. Arie Vonk, Piet F.M. Verdonschot

Supplementary material 1

Water temperature was measured every ten minutes during 55 days between May and July 2016 with HOBO® Dissolved oxygen loggers U26-001. Data for each ditch was averaged for one loggers placed 10 cm under the water surface and one logger placed 4 cm above the bottom sediments. Additionally, chemical characteristics of the water and sediment in the ditches were determined on three moments in time. Conductivity was measured using the TDS & EC meter hold (HQ EZ1). Surface water samples were taken, filtered with 0.2µm Whatman GF/F glass fiber filters, and analyzed for total carbon, dissolved organic carbon, and total nitrogen on the elemental analyzer (Elementar Vario EL), and for orthophosphate, sulphate and chloride on the continuous flow analyzer (Skalar SAN++ system). The top 2 cm of four sediment cores were pooled, sieved through 500 µm, stored in a freezer at -22 °C, and milled. The organic matter content was determined after heating dry sediment samples at 550 °C for 4 h. Homogenized portions of 200 mg dry sediment were digested with 4 mL HNO3 (65 %) and 1 mL H2O2 (30 %), using the microwave. Digestates were diluted and phosphorus concentrations were determined by ICP (Perkin Elmer ICP-OES 8000). A homogenized portion of dry sediment was used to determine carbon and nitrogen content, using the elemental analyzer as described above.

Oxygen	Water column							Sediment		
Benthic layer anoxic (%)	Water temp (°C)	Conductivity (µS/cm)	Tot C (mg/L)	DOC (mg/L)	Tot N (mg/L)	PO^{3-}_4 (mg/L)	SO^{2-}_4 (mg/L)	Cl (mg/L)	C:N ratio	Tot P (mg/g)	Organic matter (%)
0.3	19.7±2.2	372±131	45.6±1.5	20.6±7.9	1.4 ±0.3	0.05±0.03	14.7±2.8	28.6±13.6	12.4±6.3	0.3±0.1	5.4±1.7
4.0	19.2±2.2	404±76	44.2±6.3	18.1±5.0	1.3 ±0.1	0.03±0.01	14.4±6.4	34.7±7.8	13.0±5.5	0.2±0.1	4.3±1.0
7.2	18.3±2.1	262±31	34.5±8.3	10.5±1.6	0.9 ±0.4	0.05±0.03	1.6±0.3	12.3±0.6	15.3±1.1	0.4±0.3	10.7±6.9
10.7	19.0±2.3	204±33	31.6±4.7	12.6±1.0	0.8 ±0.1	0.03±0.01	1.2±0.3	11.7±0.7	15.4±1.1	0.3±0.3	7.8±7.7
20.4	18.6±2.1	226±6	45.9±5.2	22.8±7.5	1.5 ±0.2	0.07±0.00	4.5±1.7	7.7±1.0	15.3±3.7	1.1±0.3	26.3±2.5
37.5	19.0±2.2	365±43	58.6±29.5	33.1±27.6	2.4 ±1.2	0.07±0.04	10±3.9	28.5±14.8	14.5±4.9	0.6±0.1	13.7±0.6
38.0	19.1±1.9	395±70	48.6±4.7	19.3±3.5	1.3 ±0.1	0.07±0.09	8.9±4.7	31.5±7.0	14.8±5.2	1.1±0.4	20.5±8.5
47.3	18.9±2.3	269±37	65.8±12.1	43.8±8.0	2.6 ±0.3	0.09±0.05	8.3±4.3	9.0±1.9	14.9±4.4	0.9±0.5	25.3±22
55.1	18.4±2.4	223±10	45.7±2.1	25.4±1.0	1.5 ±0.0	0.07±0.09	7.2±1.2	7.7±0.2	14.6±3.8	0.8±0.1	26.1±1.4
57.8	17.8±2.1	273±39	37.3±7.1	11.5±1.7	0.9 ±0.3	0.08±0.01	4.5±0.9	10±0.6	13.3±4.0	0.4±0.2	7.1±4.0
58.5	19.5±2.1	386±104	56.1±11.2	30.1±12.8	2.0 ±0.7	0.05±0.05	13.2±10.4	29.2±7.5	13.6±7.5	0.6±0.1	11.2±0.5
59.4	18.5±1.9	248±28	43.7±10.3	18.4±6.2	1.1 ±0.2	0.04±0.05	2.6±1.5	5.2±1.3	14.5±4.2	0.9±0.9	22.2±23.8
69.1	17.7±1.9	226±12	33.0±2.5	10.2±2.3	0.8 ±0.1	0.09±0.03	1.5±0.2	7.8±0.1	13.9±1.1	0.4±0.3	7.3±5.0
70.7	19.4±2.3	239±44	39.3±5.3	21.1±6.5	1.3 ±0.3	0.04±0.05	3.3±4.0	16.2±3.7	15.4±4.6	0.5±0.2	16.8±15.7
91.4	19.2±2.3	276±50	39.1±9.2	15.5±5.2	0.9 ±0.3	0.04±0.04	4.1±2.3	13.0±1.9	16.6±4.8	1.1±0.1	53.9±4.3

Supplementary Table S1: Overview of the physicochemical characteristics of the water column and sediment in each ditch (water temperature n = 17280 readings, other parameters n = 3 samples, mean ± sd).

Supplementary material 2

Taxa	Family	Functional feeding group			Food	Detri-tivore
		CG	SH	FI	DET	
Crangonyx pseudogracilis	Amphipoda	0	1	0	1	1
Gammarus pulex	Amphipoda	1	1	0	1	1
Argyroneta aquatica	Aranea	0	0	0	0	0
Sphaeridae	Bivalvia	0	0	1	1	1
Odontomyia sp.	Brachycera	1	1	0	1	1
Ablabesmyia sp.	Chironomidae[1]	0	0	0	0	0
Chironomus sp.	Chironomidae[1]	1	0	1	1	1
Cladopelma gr. *lateralis*	Chironomidae[1]	1	0	0	1	1
Clinotanypus nervosus	Chironomidae[1]	0	0	0	0	0
Cricotopus sp.	Chironomidae[1]	1	0	0	1	1
Endochironomus sp.	Chironomidae[1]	0	0	1	1	1
Microtendipes sp.	Chironomidae[1]	1	0	0	1	1
Polypedilum sp.	Chironomidae[1]	1	0	1	1	1
Procladius sp.	Chironomidae[1]	0	0	0	0	0
Psectrocladius gr. *limbatellus/sordidellus*	Chironomidae[1]	1	0	0	1	1
Psectrocladius gr. *platypus/obvius*	Chironomidae[1]	1	0	0	0	0
psectrotanypus varius	Chironomidae[1]	0	0	0	0	0
Tanypus kraatzi	Chironomidae[1]	1	0	0	1	1
Zavrelia marmorata	Chironomidae[1]	1	0	0	1	1
Agabus bipustulatus	Coleoptera	0	1	0	0	0
Graphoderus cinereus	Coleoptera	0	0	0	0	0
Haliplus flavicollis	Coleoptera	0	1	0	0	0
Hydaticus seminiger	Coleoptera	0	0	0	0	0
Hydaticus transversalis	Coleoptera	0	0	0	0	0
Hydrochara caraboides	Coleoptera	0	1	0	1	1
Hyphydrus ovatus	Coleoptera	0	0	0	0	0
Laccobius minutus	Coleoptera	1	1	0	1	1
laccophilus hyalinus	Coleoptera	1	1	0	1	1
Laccophilus minutus	Coleoptera	1	1	0	1	1
Noterus clavicornis	Coleoptera	0	0	0	0	0
Noterus crassicornis	Coleoptera	0	0	0	0	0
Rhantus exoletus	Coleoptera	0	0	0	0	0
Agabus sp.	Coleoptera lv.[2]	0	0	0	0	0
Cybister sp.	Coleoptera lv.[2]	0	0	0	0	0
Graptodytes sp.	Coleoptera lv.[2]	0	0	0	0	0
Haliplus sp.	Coleoptera lv.[2]	0	1	0	0	0
hydrophilus sp.	Coleoptera lv.[2]	0	0	0	0	0
Hyphydrus sp.	Coleoptera lv.[2]	0	0	0	0	0
Laccophilus sp.	Coleoptera lv.[2]	0	0	0	0	0
Astacidea	Decapoda	0	1	0	1	1
Caenis horaria	Ephemeroptera	1	0	0	1	1
Caenis robusta	Ephemeroptera	1	0	0	1	1
Cloeon dipterum	Ephemeroptera	1	0	0	1	1

Anisus vortex	Gastropoda	0	0	0	1	0
Bithynia leachii	Gastropoda	0	0	1	1	1
Bithynia tentaculata	Gastropoda	0	0	1	1	1
Gyraulus albus	Gastropoda	0	0	0	0	0
Gyraulus crista	Gastropoda	0	0	0	0	0
Lymnaea stagnalis	Gastropoda	0	0	0	1	0
Physa fontinalis	Gastropoda	0	0	0	1	0
Physella acuta	Gastropoda	0	0	0	1	0
Planorbis carinatus	Gastropoda	0	0	0	1	0
Radix labiata/balthica	Gastropoda	0	0	0	1	0
Valvata piscinalis	Gastropoda	0	0	0	1	0
Corixa punctata	Heteroptera	1	0	0	1	1
Corixidae	Heteroptera	1	0	0	1	1
Cymatia coleoptrata	Heteroptera	0	0	0	0	0
Ilyocoris cimicoides	Heteroptera	0	0	0	0	0
Notonecta sp.	Heteroptera	0	0	0	0	0
Plea minutissima	Heteroptera	0	0	0	0	0
Ranatra linearis	Heteroptera	0	0	0	0	0
Sigara distincta	Heteroptera	1	0	0	1	1
Sigara falleni	Heteroptera	1	0	0	1	1
Sigara fossarum	Heteroptera	1	0	0	1	1
Sigara semistriata	Heteroptera	1	0	0	1	1
Sigara striata	Heteroptera	1	0	0	1	1
Erpobdella octoculata	Hirudinea	0	0	0	0	0
Hydracarina	Hydracarina	0	0	0	0	0
Asellus aquaticus	Isopoda	1	1	0	1	1
Proasellus meridianus	Isopoda	1	1	0	1	1
Sialis lutaria	Megaloptera	0	0	0	0	0
Anophelinae	Nematocera	1	0	0	1	1
Ceratopogonidae	Nematocera	0	0	0	0	0
Chaoborus sp.	Nematocera	0	0	0	0	0
Dixella sp.	Nematocera	1	0	1	1	1
Aeschnidae	Odonata	0	0	0	0	0
Coenagrionidae	Odonata	0	0	0	0	0
Oligochaeta	Oligochaeta	1	0	0	1	1
Tricladida	Platyhelminthes	0	0	0	0	0
Agrypnia pagetana	Trichoptera	0	1	0	0	0
Athripsodes aterrimus	Trichoptera	0	1	0	0	0
Holocentropus picicornis	Trichoptera	0	0	0	0	0
Oecetis struckii	Trichoptera	0	1	0	0	0
Triaenodes bicolor	Trichoptera	0	1	0	1	1
Tricholeiochiton fagesi	Trichoptera	0	0	0	0	0

Supplementary Table S2: List of detritivores based on combination of functional feeding group (CG = collector gatherer, SH = shredder, FI = filter feeder) and food (DET = detritus or dead plant). Information based on Tachet (2010). 1) Additional information Chironomidae based on Moller Pilot (2009, 2013). 2) Coleoptera larvae (lv) were assessed separate from adults for feeding groups (not for richness).
